# Maternal Low-Protein Diet During Nursing Leads to Glucose–Insulin Dyshomeostasis and Pancreatic-Islet Dysfunction by Disrupting Glucocorticoid Responsiveness in Male Rats

**DOI:** 10.3390/biology13121036

**Published:** 2024-12-11

**Authors:** Paulo Cezar de Freitas Mathias, Aline Milena Dantas Rodrigues, Patrícia Cristina Lisboa, Rosiane Aparecida Miranda, Ananda Malta, Tatiane Aparecida Ribeiro, Luiz Felipe Barella, Ginislene Dias, Thalyne Aparecida Leite Lima, Rodrigo Mello Gomes, Egberto Gaspar de Moura, Júlio Cezar de Oliveira

**Affiliations:** 1 Laboratory of Secretion Cell Biology, Department of Biotechnology, Genetics and Cell Biology, State University of Maringa, Maringá 87020-900, Brazil; pmathias@uem.br (P.C.d.F.M.); nandamalt@hotmail.com (A.M.); tatianeribeiro2@hotmail.com (T.A.R.); lfbarella@gmail.com (L.F.B.); 2 Research Group on Perinatal Programming of Metabolic Diseases: DOHaD Paradigm, Laboratory of Metabolic and Cardiovascular Diseases, Health Education and Research Center (NUPADS), Institute of Health Sciences, Federal University of Mato Grosso, University Campus of Sinop, Sinop 78556-264, Brazil; aline.ar60@gmail.com (A.M.D.R.); ginislenemiranda@gmail.com (G.D.); thalyneleite@hotmail.com (T.A.L.L.); 3 Laboratory of Endocrine Physiology, Department of Physiological Sciences, State University of Rio de Janeiro, Rio de Janeiro 20550-013, Brazil; pclisboa.uerj@gmail.com (P.C.L.); roapmiranda@yahoo.com.br (R.A.M.); egmoura3@gmail.com (E.G.d.M.); 4 Laboratory of Endocrine Physiology and Metabolism, Institute of Biological Sciences, Federal University of Goiás, Goiânia 74690-900, Brazil; gomesrn@ufg.br

**Keywords:** lactation, undernutrition, glucocorticoids, insulin resistance, insulin secretion

## Abstract

Both perinatal malnutrition and elevated glucocorticoids are pivotal triggers of the growing global pandemic of metabolic diseases. Here, we studied the effects of metabolic stress responsiveness on glucose–insulin homeostasis and pancreatic-islet function in male Wistar offspring whose mothers underwent protein restriction during lactation. During the first two weeks after delivery, lactating dams were fed a low-protein (4% protein, LP group) or normal-protein diet (22.5% protein, NP group). At 90 days of age, male rat offspring were challenged with food deprivation (72 h of fasting), intracerebroventricular (icv) injection of dexamethasone (2 µL, 2.115 mmol/L) or chronic intraperitoneal injection of dexamethasone (1 mg/kg body weight/5 days). Body weight, food intake, intravenous glucose tolerance test (ivGTT) results, insulin secretion and biochemical parameters were assessed. LP rats did not display significant metabolic changes after long-term starvation (*p* > 0.05) or under the central effect of dexamethasone (*p* = 0.999). Chronic dexamethasone induced rapid hyperglycemia (~1.2-fold, *p* < 0.001) and hyperinsulinemia (NP: 65%; LP: 216%; *p* < 0.001), decreased insulin sensitivity (NP: ~2-fold; LP: ~4-fold; *p* < 0.001), reduced insulinemia (20%) and increased glycemia (35%) only in NP rats under ivGTT conditions (*p* < 0.001). Glucose and acetylcholine insulinotropic effects, as well as the muscarinic receptor antagonist response, were reduced by chronic dexamethasone only in pancreatic islets from NP rats (*p* < 0.05). The direct effect of dexamethasone on pancreatic islets reduced insulin secretion (NP: 60.2%, *p* < 0.001; LP: 33.8%, *p* < 0.001). Peripheral glucose–insulin dyshomeostasis and functional failure of pancreatic islets in LP rats, as evidenced by an impaired acute and chronic response to metabolic stress, may be due to excessive corticosterone action as a long-term consequence.

## Simple Summary

Undernutrition, especially during perinatal life, can act as critical factor on disrupting the homeostasis of body energy and glucose metabolism, which remains throughout life. In fact, when undernutrition occurs only during suckling phase it programs pups to have high insulin sensitivity in peripheral tissues, as well as pancreatic β-cells poor ability on secreting insulin. In addition, glucocorticoids are known by disrupt glucose-insulin homeostasis in humans and animal-models. Given that, herein we aimed to access the responsiveness of these undernourished rat-model to synthetic glucocorticoids dexamethasone on glucose-insulin homeostasis and pancreatic islet function. The body of data in the present article shows that rat offspring whose mothers underwent undernutrition, when nursing their pups, display a lean phenotype, glucose–insulin dyshomeostasis and functional failure of pancreatic islets (pancreatic-islets’ poor capacity on secreting insulin under the action of glucose and cholinergic signal), that is associated to the action of corticosterone in these rats. In summary, it is highlighted by failure on acute and chronic response to metabolic stress that may be due to excessive corticosterone action as a long-term consequence.

## 1. Introduction

The rapid increase in the incidence of type 2 diabetes mellitus associated with obesity has become a health problem worldwide. Maternal lifestyle and the environment, including nutrition and stress, influence the overall health of offspring [1]. However, seminal work completed nearly 35 years ago [2] and evidence from epidemiological and experimental studies demonstrated that the early-life environment, including the in utero environment experienced by the embryos, impacts health and disease risk later in life.

Caloric and/or protein dietary restriction, especially in early life, is involved in metabolic disease outcomes, as are the underlying physiological mechanisms, which are not yet well known [3,4]. Metabolic programming early in life has been reported as a key factor underlying this important pathophysiological condition. Therefore, a low-protein diet has been used as a pivotal tool to induce, in rats, metabolic dysfunctions similar to those that are diagnosed in diabetic patients with metabolic syndrome [5,6,7,8,9,10].

Opposing effects on glucose–insulin homeostasis can be induced by low protein-calorie restriction, depending on the severity, duration or period in which protein-diet restriction is applied. Specifically, glycemia, insulinemia and peripheral insulin sensitivity might increase or decrease in the bloodstream, as might endocrine pancreatic function, depending on the age of the animal [11,12,13,14].

Similarly, the effects of synthetic glucocorticoids on the maturation and/or function of the hypothalamic-pituitary-adrenal (HPA) axis [15] have been implicated in insulin resistance [16,17]. Additionally, adult rat offspring whose pregnant and/or lactating dams were chronically treated with dexamethasone and that experienced undernutrition developed insulin resistance [1,18]. In addition, humans exposed to high levels of dexamethasone [19] or cortisol [20] during fetal life have glucose intolerance and a reduced ability of the pancreas to secrete insulin, in addition to other metabolic dysfunctions.

Whereas glucocorticoids contribute to insulin resistance in both humans and rodents [21,22,23], a low-protein diet, just during the first two weeks of nursing, imprints rat progeny to high peripheral insulin sensitivity associated with functional failure of the pancreatic islets to release insulin [10,14,24,25]. However, there are no data concerning the response of adult animals whose mothers are on a protein-restricted diet during lactation. We hypothesize that metabolism, the HPA axis and the endocrine pancreas of this early-treated rat model respond poorly to the chronic effects of synthetic glucocorticoids, since young adult offspring develop high peripheral insulin sensitivity in this model. Thus, herein, we aimed to evaluate the effects of dexamethasone on glucose–insulin homeostasis and the ability of pancreatic islets to secrete insulin in a well-established model of rat offspring from mothers fed a low-protein diet in the suckling phase.

## 2. Materials and Methods

### 2.1. Ethical Approval

All the experimental protocols were approved by the Ethics Committee for Animal Use and Experiments of the State University of Maringá (CEUA/UEM; process number 8981290814), which adheres to Brazilian Federal Law number 11.794/2008. Our study complies with the animal ethics checklist as described by [26].

### 2.2. Animal Groups and Dietary Manipulation

The study was performed using adult Wistar rats. Throughout the experimental period, the rats were kept under controlled conditions of temperature (22 ± 2 °C) and humidity (60 ± 5%) with a 12 h light cycle (07:00 a.m.–07:00 p.m.); water and food were available ad libitum.

After delivery, lactating dams (8 female rats in each group) were fed a standard laboratory diet containing 22.5% protein (Nuvital^®^, Curitiba, PR, Brazil) during lactation or a low-protein diet with 4% protein content during the first 14 days of lactation, after which they returned to a normal laboratory diet for the remaining third of lactation. The number of calories in the low-protein diet was the same as that in the normal-protein diet [14]. Thus, the rats were randomized into two different groups (rat offspring from dams fed a normal-protein (NP) or low-protein (LP) diet). Each lactating dam was housed with 6 male pups throughout the lactation period. At 21 days old, the pups were weaned and thereafter fed a normal diet ad libitum until they reached 90 days old, at which time the rats were analyzed (n = 48 male rat offspring from 8 litters for each group). Because sex differences in insulin levels and glucose tolerance have been previously observed in rat offspring fed on a protein-restricted diet early in life [27], after weaning, only male rat offspring were used in the study.

### 2.3. Effects of Starvation Challenge on Hormone Parameters and Intravenous Glucose Tolerance Test (ivGTT) Results

To test the acute action of a stressful environment, we induced food deprivation to study the response of the HPA axis by assessing corticosteronemia and glucose–insulin homeostasis, measuring glycemia and insulinemia during the intravenous glucose tolerance test (ivGTT). For this experiment, a cohort of 90-day-old rat offspring (n = 8 rats from 8 litters) underwent 72 h of fasting. After that, the rats were subjected to the ivGTT (as described in the following section) for blood collection (350–400 µL) through the right jugular vein and subsequent plasma glucose, insulin and corticosterone assessment.

### 2.4. Intracerebroventricular (icv) Cannula Implantation Surgery and Assessment of the Central Effect of Dexamethasone

Under thiopental anesthesia (45 mg/kg body weight, intraperitoneally), another cohort of 90-day-old rat offspring (n = 16 rats from 8 litters) was transferred to a stereotaxic apparatus (Insight^®^, Ribeirão Preto, SP, Brazil). To allow the injection of small volumes into the right lateral ventricle, a guide cannula of stainless steel measuring 0.6 mm in diameter was implanted according to the Paxinos and Watson coordinates [28]. After the head was shaved, an incision was made along the midline of the head, and the subcutaneous tissues were removed. A marking was then made on the left parietal bone according to the coordinates, and a drill was used to make an opening through which a guide cannula was introduced. The cannula was inserted and positioned 1 mm above the right lateral ventricle. The cannula was fixed in place with self-curing acrylate glue.

At the end of surgery, to assess the central effect of dexamethasone, each rat was kept in an individual cage for 5 days of surgery recovery time. The cannula placement was subsequently tested for a dipsogenic response induced by angiotensin II (2 µL of 10^−6^ mol/L solution) infusion; rats that drank less than 5 mL within 15 min after treatment were excluded from the study [29,30].

The rats were deprived of food for 12 h (7:00 a.m.–7:00 p.m.) while retaining free access to water. The icv injection of saline (NaCl, 0.9%, n = 8 rats) or dexamethasone (2.115 mmol/L, n = 8 rats) was performed at 7:00 p.m. [31]. After that, the rats were fed standard chow, and food intake was determined by measuring the difference between the weight of the chow given and the weight of chow at the end of the two different periods, at 4 h (11:00 p.m.) and 12 h (7:00 a.m.) after the icv injection, as previously reported [32]. The food intake is presented relative to 100 g of body weight.

### 2.5. Measurement of the Fat Pad Stores

At the end of each experimental procedure, the rat offspring (n = 48 rats from 8 litters) were euthanized, and representative fat pad stores (retroperitoneal, epididymal, mesenteric and subcutaneous inguinal white adipose tissues) were removed and weighed. To assess the body composition phenotype, the adiposity index was used, where all the representative white adipose tissues were summed and then divided by 100 g of each rat’s body weight. The data are presented as g per 100 g of body weight.

### 2.6. Effects of Chronic Dexamethasone Challenge on Biochemical and Metabolic Parameters

The effect of chronic dexamethasone treatment on the metabolic responsiveness of rats was studied in another cohort of 90-day-old rat offspring (n = 24 rats from 8 litters) by evaluating the following parameters: plasma levels of corticosterone and adrenocorticotrophic hormone (ACTH), which are used to study the HPA axis response; ivGTT, which is used to study glucose-insulin homeostasis; glucose-induced insulin secretion, acetylcholine (ACh)-potentiated insulin secretion and the M_3_ muscarinic ACh receptor (M_3_mAChR) response, which are used to study the pancreatic islet function.

The rats were intraperitoneally injected with dexamethasone (1 mg/kg body weight, n = 12) for 5 consecutive days [33], while the counterpart rats received an intraperitoneal injection of saline solution (0.9%, NaCl, n = 12). Food intake and body weight gain during dexamethasone treatment were evaluated daily. Some of these rats subsequently underwent an ivGTT (n = 8 rats from 8 litters), whereas others (n = 4 rats from 4 litters) were used to study pancreatic islet function, as described in the following section.

### 2.7. Intravenous Glucose Tolerance Test (ivGTT)

The surgical procedure for silicone cannula implantation into the right jugular vein, as well as the ivGTT, was performed as previously described [14]. After overnight fasting (07:00 p.m.–07:00 a.m.), a glucose load (1 g/kg of body weight) was infused through the cannula into the bloodstream of conscious rats. Blood samples (350–400 µL) were collected immediately before the glucose load (0 min) and at 5, 15, 30 and 45 min.

Blood samples were taken at the times indicated above, and the resulting plasma samples were stored at −20 °C for further analyses. The glucose concentration was determined via the glucose oxidase method [34] using a commercial kit (*Gold* Analisa^®^, Belo Horizonte, MG, Brazil). The levels of insulin were determined via radioimmunoassay [35] with a gamma counter (Wizard^2^ Automatic Gamma Counter, TM-2470, PerkinElmer^®^, Shelton, CT, USA). The other reagents used were human insulin as a standard, an anti-rat insulin antibody (Sigma-Aldrich^®^, St. Louis, MO, USA) and ^125^I-labeled recombinant human insulin (PerkinElmer^®^, Shelton, CT, USA). The intra- and interassay coefficients of variation for insulin were 12.2% and 9.8%, respectively. The detection limit of insulin was 1.033 pmol/L.

### 2.8. Insulin Sensitivity Index

The insulin sensitivity index (ISI), which is a reasonable approximation of whole-body insulin sensitivity [36], is one of the methods used to measure whole-body insulin sensitivity. To calculate the ISI, we performed the following calculation: ISI = 10^4^/√ [(fasting glycemia) × (fasting insulinemia) × (AUC∆_Glycemia_ × AUC∆_Insulinemia_)], as previously described [32].

The homeostatic model assessment of insulin resistance (HOMA-IR, calculated as (fasting glycemia) × (fasting insulinemia)/22.5) was also used to assess body insulin sensitivity [37].

### 2.9. Pancreatic Islet Isolation

Around 1200 pancreatic islets were isolated from each rat pancreas (n = 4 rats from 4 litters) via a collagenase technique as described previously [38]. At 90 days old, the rats were decapitated, and the abdominal wall was opened for injection of 8 mL of Hanks’ buffered saline solution (HBSS) into the common bile duct, as previously described [38]. The pancreas, swollen with collagenase solution, was quickly excised and incubated at 37 °C in a glass beaker for 17–18 min for the NP rats or 11–12 min for the LP rats. The suspension was then discarded, and the pancreas was washed with HBSS for 3 continuous washes. The islets were collected with the aid of a stereomicroscope.

### 2.10. Stimulation of Insulin Secretion

To adapt the isolated islets to a baseline glucose concentration (5.6 mmol/L), the islets (4 islets per well, in a 24-well plate) were preincubated for 60 min in 1 mL of normal Krebs–Ringer solution [38]. To perform the study of pancreatic islet incubation, a pool of 16 isolated pancreatic islets (4 islets per well) was used. All the isolated pancreatic islets were obtained from 4 rats from 4 different litters in each group. All the studies of pancreatic islet incubation were repeated twice.

Increasing glucose concentrations [(mmol/L): 5.6, 8.3, 11.1, 16.7, 20.0 and 24.0] as well as ACh concentrations [(µmol/L): 0.1, 1, 10, 100 and 1000] in the presence of 8.3 mmol/L glucose were used to evaluate glucose-induced insulin secretion and the cholinergic response in pancreatic islets from NP and LP rats, of which some were previously chronically treated with dexamethasone. In addition, the M_3_mAChR response in pancreatic islets from NP and LP rats treated with or without dexamethasone was assessed by using 4-diphenylacetoxy-N-methylpiperidine methiodide (4-DAMP, 100 µmol/L), a selective M_3_mAChR antagonist, in the presence of 8.3 mmol/L glucose and 10 µmol/L ACh.

To study the direct effect of dexamethasone, following preincubation, a cohort of isolated pancreatic islets from NP and LP rats that were not chronically treated with dexamethasone were incubated for an additional 60 min in Krebs–Ringer solution containing 16.7 mmol/L glucose and/or 16.7 mmol/L glucose plus dexamethasone (µmol/L: 0; 1; 2; 4; 8; 16).

All the drugs described above for studying pancreatic islet function were purchased from Sigma-Aldrich (Sigma-Aldrich^®^, St. Louis, MO, USA).

### 2.11. Hormone Plasma Levels

Commercial ELISA kits were used according to the manufacturer’s recommendations to measure the plasma levels of corticosterone (catalog number ADI-900-097, Enzo^®^ Life Sciences, Plymouth Meeting, PA, USA) and ACTH (catalog number MBS035803, MyBioSource^®^, San Diego, CA, USA).

The intra- and interassay coefficients of variation were 7.7% and 9.7%, respectively, for corticosterone and 3.7% and 4.7%, respectively, for ACTH. The hormone level detection limits were 74.46 pmol/L for corticosterone and 0.22 pmol/L for ACTH.

### 2.12. Statistical Analyses

The results are presented as the means ± SEMs and were subjected to the Shapiro–Wilk normality test. Gaussian distribution data were subjected to parametric tests (Student’s *t* test or one-way analysis of variance (one-way ANOVA), followed by Tukey’s multiple comparisons post hoc test). The value of *p* < 0.05 was considered statistically significant. Tests were performed using GraphPad Prism version 7.0 for Windows (GraphPad Software Inc., San Diego, CA, USA).

## 3. Results

### 3.1. Biometric Profile of Offspring from Maternal Rats Fed a Low-Protein Diet

At 90 days of age, LP rats presented a reduced body weight (17.3%, NP rats: 376.2 ± 4.16 g versus LP rats: 311.0 ± 2.86 g; *p* < 0.001, n = 48), and the adiposity index was 23% lower (NP rats: 2.84 ± 0.11 g/100 g of body weight versus LP rats: 2.19 ± 0.06 g/100 g of body weight; *p* < 0.001, n = 48).

### 3.2. Body Weight and Food Intake During Dexamethasone Treatment

**Figure 1:**
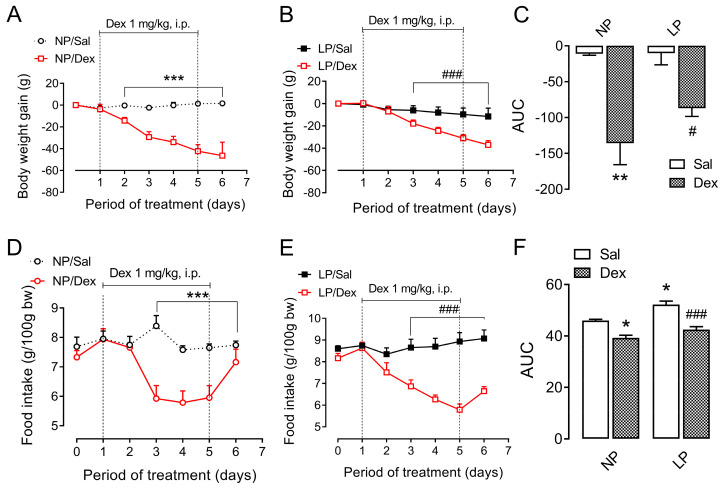
Body weight and food intake assessment throughout the chronic dexamethasone treatment. The data are presented as the means ± SEMs of 12 rats from 8 litters. Body weight evolution of NP (**A**) and LP (**B**) rats and food intake of NP (**D**) and LP (**E**) rats during dexamethasone treatment. The significant differences among the points in the curves (**A**,**B**,**D**,**E**) were analyzed via Student’s *t* test, and the values representing the area under the curve (AUC) for body weight (**C**) and food intake (**F**) were analyzed via one-way ANOVA, followed by Tukey’s post hoc test. * *p* < 0.05, ** *p* < 0.01 and *** *p* < 0.001 denote significant differences versus NP, and ^#^ *p* < 0.05 and ^###^ *p* < 0.001 denote significant differences versus LP. NP, normal-protein diet; LP, low-protein diet.

The body weight reduction of the NP rats treated with dexamethasone was twelve times that of the NP rats not treated with dexamethasone (*p* < 0.01, Figure 1A,C). In LP rats, dexamethasone treatment reduced body weight by an eightfold greater margin (*p* < 0.05, Figure 1B,C).

Compared with control rats, NP rats treated with dexamethasone ate 14.6% less food (*p* < 0.05, Figure 1D,F). Additionally, LP rats that underwent dexamethasone treatment were hypophagic compared with LP rats that received only saline (−18.6%, *p* < 0.001, Figure 1E,F).

### 3.3. Central Effect of Dexamethasone on Food Intake During the Dark Cycle

**Figure 2:**
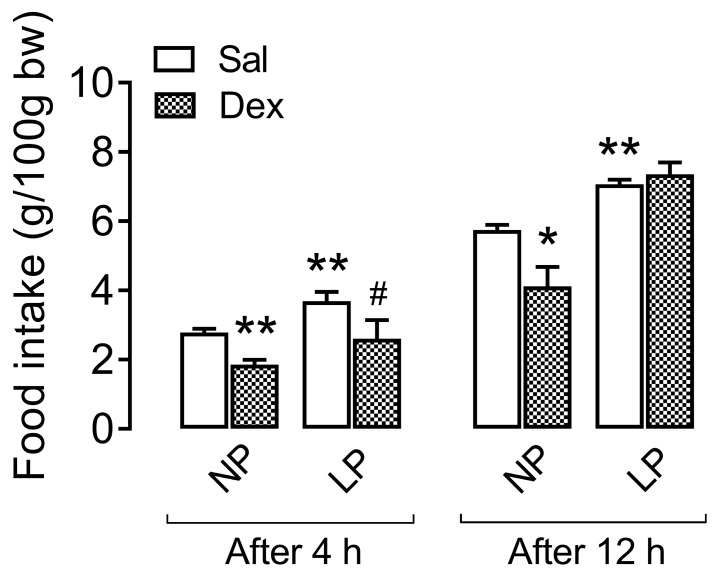
Ad libitum food intake during the dark cycle after icv injection of dexamethasone. The data are presented as the means ± SEMs of 12 h fasted (7:00 a.m.–7:00 p.m.) 8 rats obtained from 8 litters. The values were assessed 4 h (at 11:00 p.m.) and 12 h (at 7:00 a.m.) after saline or dexamethasone intracerebroventricular (icv) injection. The significant differences were analyzed with one-way ANOVA, followed by Tukey’s post hoc test, for 4 h and 12 h. * *p* < 0.05 and ** *p* < 0.01 denotes significant differences versus NP, and ^#^ *p* < 0.05 denotes a significant difference versus LP. NP, normal-protein diet; LP, low-protein diet.

Compared with NP rats that were not treated with dexamethasone, LP rats were hyperphagic during the dark cycle at both time points of food intake assessment (Figure 2). Four hours after icv saline injection, the LP rats’ food intake was 32.6% greater (*p* < 0.01) than that of the NP rats, and 12 h after icv saline injection, it was 23% greater (*p* < 0.01) than that of the NP rats.

Compared with the respective controls, the central effect of dexamethasone induced a decrease in food intake 4 h after the icv injection of dexamethasone in NP rats (33.3%, *p* < 0.01) and LP rats (29.4%, *p* < 0.05). Interestingly, while the food intake of the NP-treated rats was reduced by 28.3% at 12 h after the intravenous injection of dexamethasone (*p* < 0.05), it was not different from that of the LP-treated rats (*p* = 0.999).

### 3.4. Biochemical Profile After Starvation Challenge

Directly comparing NP rats versus LP rats (Table 1), under 12 h fasting conditions, LP rats were normoglycemic (*p* = 0.332), hypoinsulinemic (−35%, *p* < 0.001) and hypercorticosteronemic (+105.6%, *p* < 0.001) and presented lower levels of ACTH (63.4%, *p* < 0.01, Table 2) than did NP rats.

Compared with NP rats, 12 h fasted LP rats presented a 39.3% reduction in HOMA-IR and an 80.2% increase in ISI values (*p* < 0.01).

After 72 h of fasting, glycemia did not change in the NP or LP rat group. On the other hand, insulinemia was lower in both the NP (3.6-fold, *p* < 0.01; Table 1) and LP (2.3-fold, *p* < 0.01; Table 1) rats than in the 12 h fasted rats. The HOMA-IR decreased by 73.3% in the NP rats fasted for 72 h (*p* < 0.001), whereas in the LP rats, the long fasting condition reduced this parameter by 62.6% (*p* < 0.05). The ISI values increased in NP rats by 70.4% (*p* < 0.001, Table 1) and by 62.3% in the LP rats (*p* < 0.05, Table 1) than in their respective controls after 12 h of fasting.

Compared with those of 12 h fasted NP rats, the blood levels of corticosterone were greater in the NP rat group (Table 1). While corticosteronemia was approximately 32.7% greater in NP rats after 72 h of fasting (*p* < 0.05), it did not change in LP rats (*p* = 0.999).

### 3.5. Biochemical Profile After Chronic Dexamethasone Challenge

As shown in Table 2, treatment with dexamethasone induced hyperglycemia in both the NP rats (+20.6%, *p* < 0.001) and LP rats (+22.4%, *p* < 0.001), as well as hyperinsulinemia in the NP rats (+65.2%, *p* < 0.001) and LP rats (+215.6%, *p* < 0.001). Moreover, in comparison with the controls, dexamethasone treatment increased the HOMA-IR by 1.9-fold in the NP rats and by 3.9-fold in the LP rats (*p* < 0.001, Table 2). ISI values were reduced by 40.3% in NP rats (*p* < 0.01, Table 2) and by 42.8% in LP rats (*p* < 0.05, Table 2) in comparison to their respective controls.

Interestingly, the plasma levels of ACTH decreased by 44.1% in the NP-treated rats (*p* < 0.01, Table 2), whereas there was no change in the LP rats (*p* = 0.200, Table 2). Additionally, with dexamethasone treatment, the blood corticosterone concentration decreased by 36.2% in the NP rats (*p* < 0.001, Table 2) and by 45.8% in the LP rats (*p* < 0.05, Table 2).

### 3.6. Insulin Homeostasis After Metabolic-Stress Challenge

**Figure 3:**
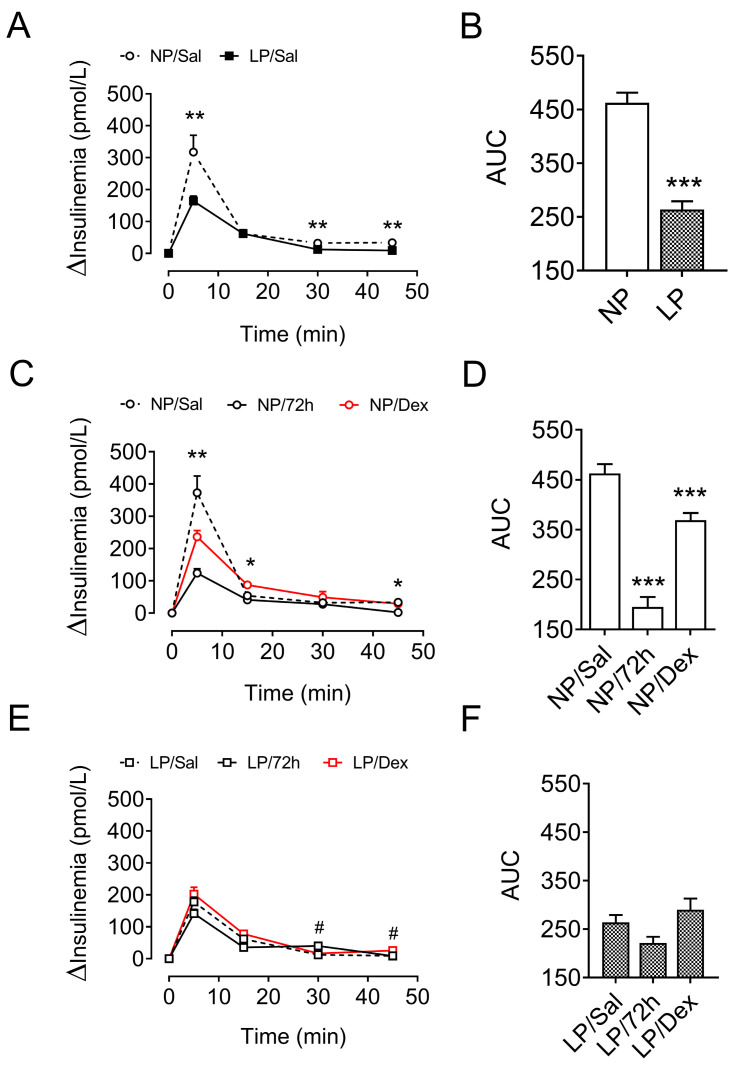
Variation in plasma insulin levels during the intravenous glucose tolerance test (ivGTT). The data are presented as the means ± SEMs of 8 rats from 8 litters. Plasma insulin levels of NP and LP rats without stress (**A**,**B**) and of NP (**C**,**D**) and LP (**E**,**F**) rats after 72 h of starvation and/or chronic dexamethasone challenge. The significant differences were analyzed with Student’s *t* test (**A**,**B**) or one-way ANOVA, followed by Tukey’s post hoc test (**C**–**F**). * *p* < 0.05, ** *p* < 0.01 and *** *p* < 0.001 denote significant differences versus NP/Sal rats, and ^#^ *p* < 0.05 denotes significant differences versus LP/Sal rats. NP, normal-protein diet; LP, low-protein diet.

As expected, LP rats presented a lower increase in plasma insulin during the ivGTT than did NP rats (43%, *p* < 0.001, Figure 3A,B).

Under the long-term starvation challenge, the increase in plasma insulin during the ivGTT in the NP rats was reduced by 57.8% (*p* < 0.001, Figure 3C,D), whereas the LP rats did not exhibit any change in plasma insulin levels (*p* = 0.171, Figure 3E,F). In addition, while chronic treatment with dexamethasone decreased insulinemia by 20.3% during the ivGTT in the NP rats (*p* < 0.05, Figure 3C,D), it was not different in the LP rats compared with the control rats (*p* = 0.390, Figure 3E,F).

### 3.7. Glucose Homeostasis After Metabolic-Stress Challenge

**Figure 4:**
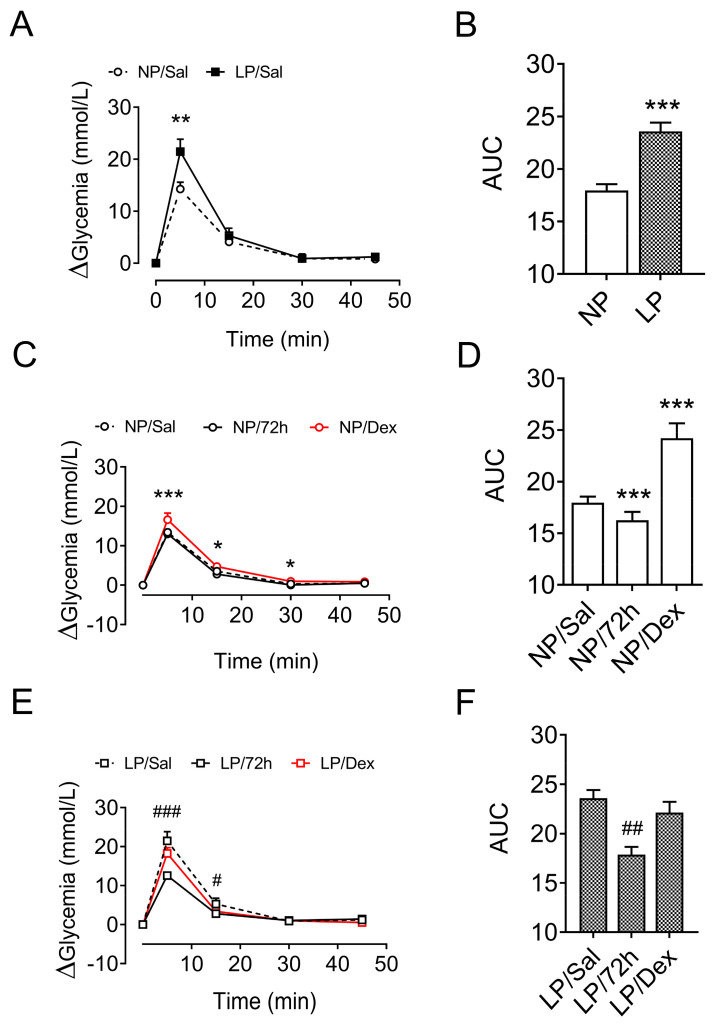
Variation in plasma glucose levels during the intravenous glucose tolerance test (ivGTT). The data are presented as the means ± SEMs of 8 rats from 8 litters. Plasma glucose levels of NP and LP rats without stress (**A**,**B**) and of NP (**C**,**D**) and LP (**E**,**F**) rats after 72 h of starvation and/or chronic dexamethasone challenge. The significant differences were analyzed with Student’s *t* test (**A**,**B**) or one-way ANOVA, followed by Tukey’s post hoc test (**C**–**F**). * *p* < 0.05, ** *p* < 0.01 and *** *p* < 0.001 denote significant differences versus NP/Sal rats, and ^#^ *p* < 0.05, ^##^ *p* < 0.01 and ^###^ *p* < 0.001 denote significant differences versus LP/Sal rats. NP, normal-protein diet; LP, low-protein diet.

The increase in glucose content during the ivGTT was 31.3% greater in the 12 h fasted LP rats than in the 12 h fasted NP rats (*p* < 0.001, Figure 4A,B).

Compared with the 12 h fasted NP rats, the 72 h fasted NP rats presented similar increases in glucose during the ivGTT (*p* = 0.794, Figure 4C,D). On the other hand, the increase in glucose during the ivGTT in the LP rats fasted for 72 h was 24.2% lower (*p* < 0.01, Figure 4E,F) than that of the 12 h fasted LP rats.

With respect to treatment with dexamethasone, glycemia increased 34.9% (*p* < 0.001, Figure 4C,D) during the ivGTT in the NP-treated rats compared with the glycemia of the 12 h fasted NP rats treated with saline. On the other hand, the increase in glycemia during the ivGTT in the LP rats treated with dexamethasone did not differ from the increase in glycemia observed in the 12 h fasted LP rats that did not receive dexamethasone treatment (*p* = 0.793, Figure 4E,F).

### 3.8. Effect of Chronic Dexamethasone Treatment on the Insulin Secretion of Pancreatic Islets

**Figure 5:**
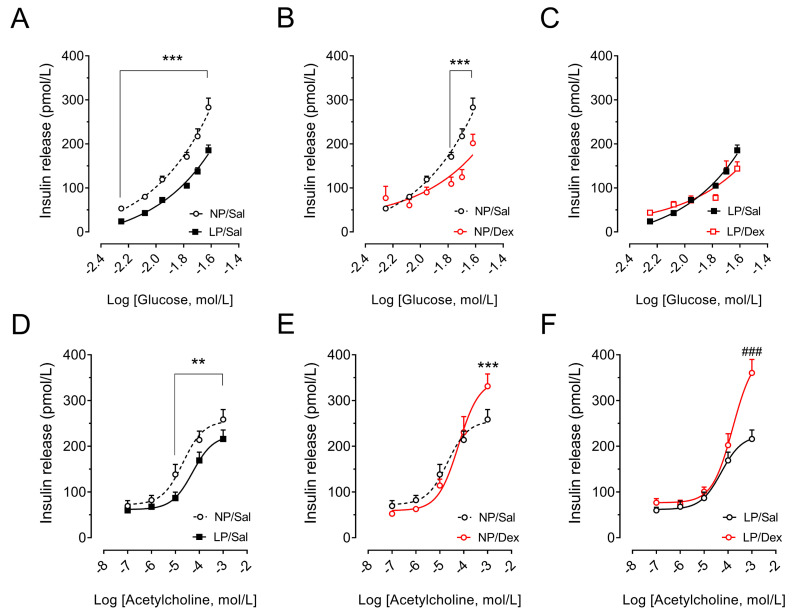
Insulin secretion from isolated pancreatic islets under the insulinotropic action of different glucose (5.6, 8.3, 11.1, 16.7, 20.0 and 24.0 mmol/L: (**A**–**C**)) and acetylcholine (0.1, 1.0, 10.0, 100.0 and 1000.0 µmol/L: (**D**,**F**)) concentrations. The symbols in the curves represent the means ± SEMs of insulin release from NP versus LP rats without stress challenges (**A**,**D**), from NP rats without stress challenges versus NP rats with chronic dexamethasone challenge (**B**,**E**) and from LP rats without stress challenges versus LP rats with chronic dexamethasone challenge (**C**,**F**). A pool of pancreatic islets (n = 32 islets) was obtained from 4 rats from 4 different litters in each experimental group. The significant differences between the groups for each glucose (**A**–**C**) and acetylcholine (**D**–**F**) concentration were determined by Student’s *t* test. ** *p* < 0.01 and *** *p* < 0.001 denote significant differences versus the NP/Sal group, and ^###^ *p* < 0.001 denote significant differences versus the LP/Sal group. NP/Sal, NP rats treated with saline; NP/Dex, NP rats treated with dexamethasone; LP/Sal, LP rats treated with saline; LP/Dex, LP rats treated with dexamethasone. NP, normal-protein diet; LP, low-protein diet.

As expected, islets from LP rats that did not receive dexamethasone treatment were less responsive in terms of insulin secretion under increasing glucose concentrations (*p* < 0.001, Figure 5A) and increasing acetylcholine concentrations (*p* < 0.05, Figure 5D) than islets from NP rats were.

Even though insulin secretion by pancreatic islets from the NP rats and LP rats treated with dexamethasone was greater than that from the controls at the baseline glucose concentration (*p* < 0.05, Figure 5B,C), it was not sustained at higher glucose concentrations. While dexamethasone treatment led the pancreatic islets from the NP rats to secrete less insulin under increasing glucose concentrations (*p* < 0.001, Figure 5B), it did not affect the ability of the pancreatic islets from the LP rats to secrete insulin (*p* > 0.05, Figure 5C).

The cholinergic response to lower and moderately high acetylcholine concentrations was reduced in the NP rats but was not affected in the LP rats treated with dexamethasone. However, the cholinergic response was increased by 28% in pancreatic islets from NP rats (*p* < 0.001, Figure 5E) and by 71.7% in those from LP rats (*p* < 0.001, Figure 5F) under the effect of a supraphysiological concentration (1000 µmol/L) of acetylcholine.

### 3.9. Assessment of Muscarinic Receptor Action and Direct Effects of Dexamethasone on Insulin Secretion in Pancreatic Islets from Rats That Were Not Chronically Treated with Dexamethasone

**Figure 6:**
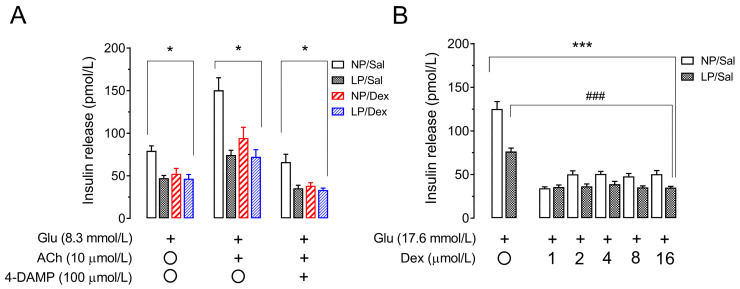
Insulin secretion from isolated pancreatic islets under the action of a selective antagonist of the insulinotropic subtype 3 muscarinic acetylcholine receptor (M_3_mAChR) (**A**) and different dexamethasone concentrations (1, 2, 4, 8 and 16 µmol/L) (**B**). The data are presented as the means ± SEMs of insulin release. A pool of pancreatic islets (n = 32 islets) was obtained from 4 rats from 4 different litters in each experimental group. The significant differences between the groups for each insulin secretagogue agent (glucose, 8.3 mmol/L; acetylcholine, 10 µmol/L; or 4-DAMP, 100 µmol/L) and dexamethasone in the presence of 16.7 mmol/L glucose were determined by one-way ANOVA, followed by Tukey’s post hoc test. * *p* < 0.05, *** *p* < 0.001 denote significant differences between each of the groups (LP/Sal, NP/Dex and LP/Dex) versus the NP/Sal group with each of the treatments (Glu, ACh and 4-DAMP) and for all the dexamethasone concentrations versus 16.7 mmol/L glucose-induced insulin secretion in the NP/Sal islets. ^###^ *p* < 0.001 denotes a significant difference between the dexamethasone concentrations and 16.7 mmol/L glucose-induced insulin secretion in LP/Sal islets. NP/Sal, NP rats treated with saline; NP/Dex, NP rats treated with dexamethasone; LP/Sal, LP rats treated with saline; LP/Dex, LP rats treated with dexamethasone. NP, normal-protein diet; LP, low-protein diet. Glu, glucose; ACh, acetylcholine; 4-DAMP, 4-diphenylacetoxy-N-methylpiperidine methiodide; Dex, dexamethasone; + indicates the presence or absence ◯ of insulin secretagogues (ACh and 4-DAMP) or dexamethasone.

In terms of insulin secretion from the NP/Sal, the pancreatic islets from the LP/Sal group secreted less insulin under the effects of 8.3 mmol/L glucose (40.25%, *p* < 0.01, Figure 6A), 16.7 mmol/L glucose (38.85%, *p* < 0.001, Figure 6B), 10 µmol/L acetylcholine (50.48%, *p* < 0.001, Figure 6A) and 100 µmol/L 4-DAMP (46.58%, *p* < 0.001, Figure 6A).

Interestingly, insulin secretion by pancreatic islets from NP rats treated with dexamethasone was similar to that observed in islets from LP rats that did not receive dexamethasone treatment under all the conditions assessed (*p* > 0.05, Figure 6A). On the other hand, treatment with dexamethasone did not affect the ability of pancreatic islets from LP rats to secrete insulin (*p* > 0.05, Figure 6A) under any of the studied conditions.

In turn, compared with that of pancreatic islets from NP rats, insulin secretion under the action of 16.7 mmol/L glucose was reduced by 36.3% in pancreatic islets from LP rats (*p* < 0.001, Figure 6B). The direct effect of 1 µmol/L dexamethasone on the isolated pancreatic islets reduced insulin secretion by 60.2% in the islets from the NP rats and by 33.8% in the islets from the LP rats (*p* < 0.001, Figure 6B). In addition, increasing concentrations of dexamethasone (2, 4, 8 and 16 µmol/L) reduced insulin secretion in islets from both groups in a similar manner and independently of the concentration but were less effective than 1 µmol/L dexamethasone.

Insulin secretion by pancreatic islets from NP rats treated with high concentrations of dexamethasone was similar to that displayed by pancreatic islets from LP rats in response to 16.7 mmol/L glucose (*p* > 0.05, Figure 6B).

## 4. Discussion

In the present study, we investigated the effects of glucocorticoids in a well-established model of early programming by protein restriction during lactation. Our findings show that corticosterone plays an important role in modulating pancreatic islet function and whole-body glucose homeostasis. LP rats are programmed to become less able, in general, to respond properly to stressful insults later in life.

LP rats exhibit decreased insulin secretion as well as high peripheral insulin sensitivity [10,24,39,40]. Interestingly, herein, LP rats presented hypercorticosteronemia along with reduced ACTH levels. This phenotype can be characterized as primary dysfunction of the HPA axis, suggesting increased sensitivity of the adrenal gland in LP rats. One limitation of the current study was that ACTH receptors in the adrenal cortex were not measured. At the physiological level, glucocorticoids act through a negative feedback loop that inhibits the HPA axis and then regulates their own levels in the blood [41]. Similarly, when we assessed feedback loop responsiveness via LP rats subjected to long periods of starvation, we found that HPA axis impairment in LP rats was not amplified, which corroborates the hypothesis that the HPA axis is excessively active in LP rats. However, the chronic effect of dexamethasone did not reduce ACTH levels in LP rats, indicating impaired pituitary responsiveness of the HPA axis. In other words, the capacity for corticosterone production is greater than the physiological capacity; thus, in the presence of other stressful stimuli, there is no further increase in corticosterone.

To test the hypothesis that LP rat offspring display poor pancreatic islet insulin secretion through glucocorticoid effects, we challenged LP rats, both in vivo and in vitro, with high levels of dexamethasone. LP rats secreted less insulin, which is a long-lasting effect of low-protein diet programming. In contrast, a compensatory effect of glucocorticoids was detected in vivo under fasting conditions and in vitro under the action of glucose at baseline. Nevertheless, in the NP and LP rat groups that were previously treated with dexamethasone, this compensatory effect was not observed in the ivGTT or in the isolated pancreatic islets incubated with high glucose concentrations. Moreover, while the response of pancreatic islets from NP rats to insulin secretion was strongly reduced in response to treatment with dexamethasone, it was not changed in pancreatic islets from LP rats. These observations might suggest a convergent interplay of maternal nutritional insults in the suckling period and glucocorticoid excess underlying endocrine pancreas derangement in LP rats.

With respect to the function of isolated pancreatic islets, the findings of the present study corroborate those of previous studies showing that islets from adult LP rats are less responsive to the secretion of insulin under the action of glucose and other insulinotropic compounds [10,14,24,25,40]. However, the use of dexamethasone for five consecutive days was able to decrease pancreatic islet function in NP rats similarly to that observed in LP rats. Dexamethasone treatment of pancreatic islets from LP rats did not further decrease insulin secretion. It is most likely that pancreatic islets from LP rats were programmed early to attenuate insulin-producing pathways, implicating a smaller content of insulin and thus the secretion of smaller amounts of insulin, which made them less affected by dexamethasone. We recently reported that these LP rats’ pancreatic deficiency is determined at an early age, such as during weaning [42]. Additionally, others have reported that mice overexpressing PGC-1α in pancreatic β-cells are glucose-intolerant and have a poor ability to secrete insulin, as well as presenting a reduced pancreatic β-cell mass [43]. Indeed, reduced pancreatic β-cell mass was reported in rats at the end of fetal life when their mothers were fed a low-protein diet during the last third and/or the full duration of pregnancy [44], as well as when dams were exposed to dexamethasone [45]. Similarly, humans exposed to high levels of synthetic [19] and endogenous [20] glucocorticoids when developing in utero presented glucose intolerance and low insulin secretion.

As we previously reported, LP rats display autonomous nervous system deregulation, which is implicated in reduced insulin secretion [14,24,25], even though the direct effects of glucocorticoids on these features have not been evaluated. To the best of our knowledge, we are the first to report that a maternal low-protein diet in the first two-thirds of the suckling period causes the pancreatic islets of LP rats to secrete a reduced amount of insulin, which seems to be associated with excessive action of glucocorticoids upon the pancreatic islets. Consistent with these findings, compared with the findings in NP rats that were not treated with dexamethasone, the ability of pancreatic islets from NP rats treated with dexamethasone to secrete insulin was decreased, reaching levels close to those observed in LP rats (treated with or without dexamethasone) under the action of insulin secretagogue agents. On the other hand, with the same cholinergic muscarinic treatment, no additional reduction in insulin secretion was observed in pancreatic islets from LP rats treated with dexamethasone in relation to the control.

Other mAChR subtypes as well as adrenergic receptors may be involved in the present results [25], which can be considered a limitation of our study, since these markers were not measured in pancreatic islets. Transgenic mice displaying high sensitivity to glucocorticoids in pancreatic β-cells have inhibited insulin secretion, possibly through increased activation and density of α_2_-adrenergic receptors in pancreatic β-cells [46].

In this context, the reduced responsiveness and content of M_3_mAChR associated with increased sensitivity and content of the M2 subtype of the mAChR (M_2_mAChR) in pancreatic islets from LP rats are important [25]. Herein, the magnitude of the effect of dexamethasone on insulin secretion under the action of 4-DAMP did not differ between pancreatic islets and LP rats (treated with or without dexamethasone), suggesting that pancreatic islets from LP rats support excessive action of glucocorticoids, which possibly influences the ability to secrete more insulin, even under metabolic demand.

The inhibitory effect of glucocorticoids on pancreatic β-cell insulin release has been previously shown to be associated with the action of 11 β-hydroxysteroid dehydrogenase type 1 [47,48]. In the present study, we showed that the direct effect of dexamethasone in isolated pancreatic islets from rats that were not previously treated with dexamethasone in vivo was capable of decreasing glucose-induced insulin secretion, independent of the dexamethasone concentration. This finding reinforces the concept that there is a decreased responsiveness of LP rats to high levels of glucocorticoids. Thus, the metabolic derangement of LP rats is more likely to reflect the direct and indirect cellular and/or molecular actions of excessive circulating glucocorticoid levels, which modulate the metabolic response of LP rats beginning in the early stages of life [42]. Interestingly, a recently published study reported that mouse and human pancreatic β-cells acutely treated with glucocorticoids for 48 h displayed a protective response to the direct effect of glucocorticoids by preserving their insulin secretion function through the cyclic-AMP pathway, even promoting a reduction in ion Ca^2+^ influx [49]. Regarding the direct action of glucocorticoids on the β-cell mechanisms underlying insulin secretion, isolated pancreatic islets chronically incubated for three days with corticosterone show impaired cytosolic flux of Ca^2+^ in response to glucose, which was observed to occur through the action of the glucocorticoid receptor [50].

## 5. Conclusions

In summary, our findings show that offspring from maternal rats fed a low-protein diet during lactation do not have their glucose–insulin homeostasis or pancreatic islet response affected by synthetic glucocorticoids. This supports the hypothesis that excessive glucocorticoids in LP rats strongly contribute to their metabolic impairments and reduced insulin secretion. In addition, a molecular mechanism in which glucocorticoids directly impair insulin secretion in pancreatic β-cells in this rat model is another possible way to contribute to the impairment of pancreatic physiology; further studies are necessary to reveal the precise underlying molecular mechanisms.

With respect to the health care of breastfeeding mothers, the current results reinforce the importance of nutrition during the breastfeeding period, which can change a child’s health and response to stress throughout life.

## Figures and Tables

**Table 1:** Stress effect of long starvation on glucose–insulin homeostasis and corticosterone levels of adult rat offspring suckled by dams that were fed a low-protein diet.

Biochemical Parameters	NP		LP	
	12 h	72 h	12 h	72 h
Glycemia (mmol/L)	5.20 ± 0.14	5.17 ± 0.26	5.08 ± 0.11	4.62 ± 0.39
Insulinemia (pmol/L)	29.31 ± 2.63	8.06 ± 1.78 ***	17.58 ± 2.50 **	7.58 ± 1.62
HOMA-IR	0.71 ± 0.07	0.19 ± 0.05 ***	0.43 ± 0.07 *	0.16 ± 0.03 ^#^
ISI	9.55 ± 0.52	16.27 ± 1.47 ***	17.30 ± 1.91 ***	27.91 ± 2.72 ^##^
Corticosteronemia (nmol/L)	1364.01 ± 67.27	1810.22 ± 80.31 *	1892.02 ± 123.10 **	1878.70 ± 118.50

The data are presented as the means ± SEMs of samples from 8 rats from 8 different litters. * *p* < 0.05, ** *p* < 0.01 and *** *p* < 0.001 denote significant differences versus NP after 12 h of fasting and ^#^ *p* < 0.05 and ^##^ *p* < 0.01 denote significant differences versus LP after 12 h of fasting according to one-way ANOVA followed by Tukey’s post hoc test. NP, normal-protein rat group; LP, low-protein rat group.

**Table 2:** Effects of chronic dexamethasone treatment on glucose-insulin homeostasis and the hypothalamic-pituitary–adrenal (HPA) axis response of adult rat offspring suckled by dams that were fed a low-protein diet.

Biochemical Parameters	NP		LP	
	Sal	Dex	Sal	Dex
Glycemia (mmol/L)	5.14 ± 0.11	6.20 ± 0.11 ***	5.28 ± 0.16	6.46 ± 0.10 ^###^
Insulinemia (pmol/L)	29.31 ± 2.63	48.42 ± 3.025 ***	14.52 ± 1.62 ***	45.83 ± 5.72 ^###^
HOMA-IR	0.71 ± 0.07	1.38 ± 0.12 ***	0.35 ± 0.04 ***	1.36 ± 0.08 ^###^
ISI	9.55 ± 0.52	5.70 ± 0.22 ***	17.30 ± 1.91 ***	9.89 ± 0.38 ^###^
ACTH (pmol/L)	57.83 ± 5.86	26.50 ± 6.75 **	21.17 ± 2.26 **	22.97 ± 2.94
Corticosteronemia (nmol/L)	1364.01 ± 67.27	870.5 ± 45.69 *	1892.02 ± 123.1 **	1027.82 ± 23.23 ^###^

The data are presented as the means ± SEMs of 8 samples from the offspring of the 8 different litters. * *p* < 0.05, ** *p* < 0.01 and *** *p* < 0.001 denote significant differences versus NP/Sal, and ^###^ *p* < 0.001 denotes significant differences versus LP/Sal according to one-way ANOVA followed by Tukey’s post hoc test. NP, normal-protein diet; LP, low-protein diet.

## Data Availability

The raw data supporting the conclusions of this article will be made available by the authors on request.

## Abbreviations

ACh	acetylcholine
ACTH	adrenocorticotrophic hormone
AUC	area under the curve
BSA	bovine serum albumin
4-DAMP	4-diphenylacetoxy-N-methylpiperidine methiodide
HBSS	Hanks’ buffered saline solution
HEPES	N-(2-hydroxyethyl-piperazine)-N’-(2-ethanesulfonic acid)
HOMA-IR	homeostatic model assessment of insulin resistance
HPA	hypothalamic–pituitary–adrenal
ICV	intracerebroventricular
ISI	insulin sensitivity index
ivGTT	intravenous glucose tolerance test
LP	low-protein
M_3_mAChR	subtype M3 muscarinic ACh receptor
NP	normal-protein
PGC-1α	peroxisome proliferator-activated receptor-gamma coactivator-1α
